# Efficient Optical Energy Harvesting in Self-Accelerating Beams

**DOI:** 10.1038/srep13197

**Published:** 2015-08-24

**Authors:** Domenico Bongiovanni, Yi Hu, Benjamin Wetzel, Raul A. Robles, Gregorio Mendoza González, Erwin A. Marti-Panameño, Zhigang Chen, Roberto Morandotti

**Affiliations:** 1 Institut National de la Recherche Scientifique, Université du Québec, Varennes, Québec J3X 1S2, Canada; 2 The MOE Key Laboratory of Weak-Light Nonlinear Photonics, and TEDA Applied Physics Institute and School of Physics, Nankai University, Tianjin 300457, China; 3 Benemérita Universidad Autónoma de Puebla, Puebla 72000, Mexico; 4 Department of Physics & Astronomy, San Francisco State University, San Francisco, CA 94132, USA; 5 Institute of Fundamental and Frontier Sciences, University of Electronic Science and Technology of China, Chengdu 610054, China

## Abstract

We report the experimental observation of energetically confined self-accelerating optical beams propagating along various convex trajectories. We show that, under an appropriate transverse compression of their spatial spectra, these self-accelerating beams can exhibit a dramatic enhancement of their peak intensity and a significant decrease of their transverse expansion, yet retaining both the expected acceleration profile and the intrinsic self-healing properties. We found our experimental results to be in excellent agreement with the numerical simulations. We expect further applications in such contexts where power budget and optimal spatial confinement can be important limiting factors.

Since their introduction in optics from the field of quantum mechanics^1^,^2^,^3^, Airy beams and, more generally, self-accelerating beams have received a significant amount of attention from the scientific community, from both a theoretical and an experimental point of view^4^. For instance, Airy beams have led to the generation of curved plasma channels^5^, electron self-accelerating beams^6^, photo-induced waveguides^7^ and optical light bullets^8^,^9^. In parallel, their widespread applications range from material micro-processing^10^ to optical trapping and manipulation^11^. The richness of the topic has been further highlighted in recent years by the possibility to generate self-accelerating beams propagating along any arbitrary convex trajectory, either by engineering the beam in real space^12^,^13^,^14^,^15^ or its spectral counterpart in the Fourier domain^16^. In most of the work reported to date, the pattern of the generated two-dimensional (2D, i.e., in two transverse directions) accelerating beams occupies a large area filled by several sub-lobes. While accelerating beams with reduced sub-lobes expansion have already been demonstrated, their study have actually been restricted to only a few special trajectories^17^,^18^,^19^, in which the required pattern was obtained by directly solving the related wave functions. Although a peak intensity enhancement for these confined accelerating beams is not unexpected, the fundamental issue of optimal energy efficiency has not been directly addressed, particularly in the context of arbitrary trajectories.

In this paper, we show both theoretically and experimentally that an appropriate shaping of the spatial spectra is capable of leading to the generation, the “narrowing”, and the peak intensity enhancement of 2D accelerating beams without significant degradations in both their propagation characteristics and intrinsic properties. For instance, we show that the intensity localized in the main lobe of self-accelerating beams propagating along several types of trajectories can be increased up to as much as 60% providing that an optimal shaping of the initial beam is done beforehand. Moreover, these generated beams exhibit significantly reduced tails; nevertheless they follow the original trajectories and, most importantly, they retain their self-healing properties.

## Results

### Theoretical Analysis

The propagation dynamics of a 2D linearly polarized beam propagating in free space under the paraxial condition is described by:


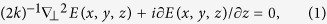


where *E* (*x, y, z*) and *k* refer to the electric field envelope and the wavenumber of the optical wave respectively, while (*x, y*) are the transverse coordinates and *z* defines the longitudinal propagation. Whenever a phase modulation *ρ*(*k*_*x*_*, k*_*y*_) is applied to an incident Gaussian beam at the Fourier plane of a spherical lens, the solution of Eq. (1) is given by:


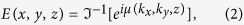


where 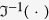 is the operator of inverse Fourier transform, the spatial spectral phase is given as *μ*(*k_x_*,*k_y_*, *z*) = – (*k_x_*^2^ + *k_y_*^2^) *z*/(2*k*) + *ρ* (*k_x_*,*k_y_*), and *k*_*x*_, *k*_*y*_ are the corresponding spatial angular frequencies. Now extending the concept of spatial phase gradient^16^ to the 2D case, the transverse coordinates of *x* and *y* are related to the gradient of *μ* (*k*_*x*_*, k*_*y*_*, z*) through the relation:


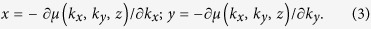


The spectral density within the area 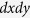, given as the inverse determinant of the Hessian matrix *Hμ*(*k*_*x*_*, k*_*y*_), shows a singularity at each propagation distance *z* (accounting for the beam trajectory) when the following condition is satisfied:


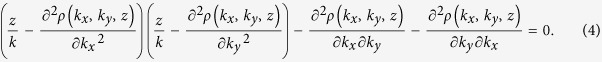


Assuming that the imposed transverse phase modulation is a separable function, i.e., *ρ*  (*k*_*x*_, *k*_*y*_) = *ρ*_*x*_(*k*_*x*_) + *ρ*_*y*_(*k*_*y*_), Eq. (4) can be reduced to:


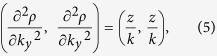


thus leading to the possibility of building a mapping relation between distance and frequency, while defining the key spatial frequencies associated with the acceleration trajectory (indicated as *k*_*xc*_(*z*) and *k*_*yc*_(*z*)). By solving Eq. (5), the key frequencies can be estimated and the beam trajectory can therefore be predicted as a parametric representation of the propagation distance *z* by means of:


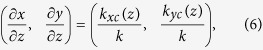


We note that the phase mask can also be designed for any desired convex beam trajectory, due to the ability of spectrum-to-distance mapping. In this case, starting from the trajectory, we can estimate the key frequencies from Eq. (6) and engineer the phase mask in a straightforward way from Eq. (5).

### Experimental Setup

In the following, we consider three typical cases of convex trajectories: a parabolic (related to the well-known Airy beam), a cubic polynomial and an exponential trajectory, whose characteristics (calculated numerically) are presented in Table 1.

As seen from Table 1, we chose the same form of phase modulation along the *x* and *y* directions so that both paths associated with the accelerating beams and corresponding key spatial frequencies are expected to project on a 45° diagonal line in the transverse plane. For the sake of clarity, we study the beam propagation characteristics along this 45° line, defining the radial position of the main hump as 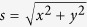 in the real space and 
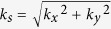
 in the spectral domain. The motivations of these design choices are in fact related to the main goal of this work. As it will be discussed later on, this specific spectral structure offers the possibility to easily increase the peak intensity of a 2D self-accelerating beam by means of a practical method based on the reshaping of the input beam.

In our experiment, an incident Gaussian beam (CW at *λ* = 633 nm, *w*_0 _= 2.45 mm) directly illuminates a spatial light modulator (SLM) located in the Fourier plane of a spherical lens (*f* = 150 mm). By applying an appropriate phase mask *ρ*(*k*_*x*_, *k*_*y*_) on the SLM, we generate a beam whose main intensity lobe follows the desired trajectory after the lens-induced Fourier transform.

**Figure 1:**
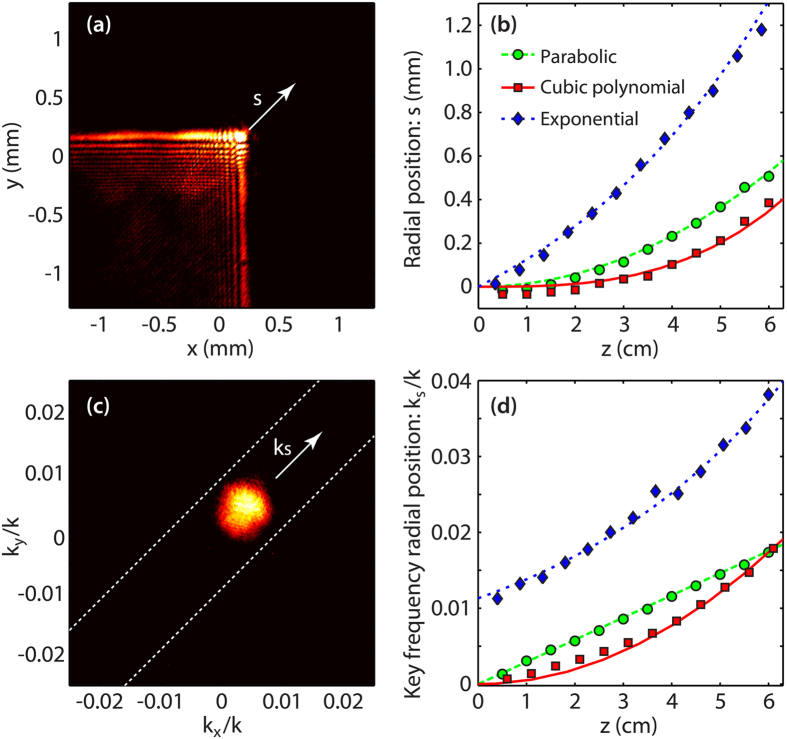
Propagation characteristics of self-accelerating beams initiated from a circular Gaussian beam. (**a**) Beam pattern obtained experimentally at *z* = 4.2 cm for the parabolic trajectory and (**c**) the spectral intensity corresponding to its main hump only. (**b**) Radial position of the main hump and (**d**) corresponding key spatial frequencies as a function of propagation distance measured for the three studied trajectories given in Table 1, where the dotted, dashed, and solid curves are from analytical results and the markers show the corresponding experimental results.

As an illustrative example, the beam intensity experimentally obtained at *z* = 4.2 cm for the case of the parabolic path is presented in Fig. 1(a). One can easily see a main lobe of high intensity surrounded by decreasing intensity side-lobes i.e., a 2D Airy beam. To confirm the reliability of our setup, we additionally measured the main lobe displacement along propagation (i.e., trajectory) of the considered accelerating beams. These results are shown in Fig. 1(b) and highlight the excellent agreement obtained between the predicted (lines) and measured (markers) convex trajectories. In the Fourier domain, the Airy beam spectrum maintains a Gaussian shape over the whole propagation range. Nevertheless, the spectral content associated with the main intensity lobe (i.e., filtering the side-lobes) corresponds to a spot of reduced size moving along the *k_s_* axis. This feature is illustrated in Fig. 1(c) where one can see that most of the energy associated to this spot (95% cut-off) is contained within a limited diagonal stripe bounded by white dashed lines. Using such an approach, we measured the locations of these spots along propagation for the three different accelerating beams, whose results are summarized in Fig. 1(d). Again, the spectral shifts of the main lobe obtained experimentally (markers) exhibit an excellent agreement with the longitudinal evolution of the key spatial frequencies (lines) predicted by Eq. (5).

### Transverse Energy Confinement through Spectral Reshaping

Owing to the fact that only the spectral components surrounding the key spatial frequencies are associated to the main lobe, the remaining spectral components are therefore linked to the beam sub-lobes of the self-accelerating beams. In numerous applications where the exact beam shape has a limited interest compared to its main lobe propagating along a given trajectory, the energy stored in the sub-lobes might therefore be considered as unwanted or even wasted.

**Figure 2:**
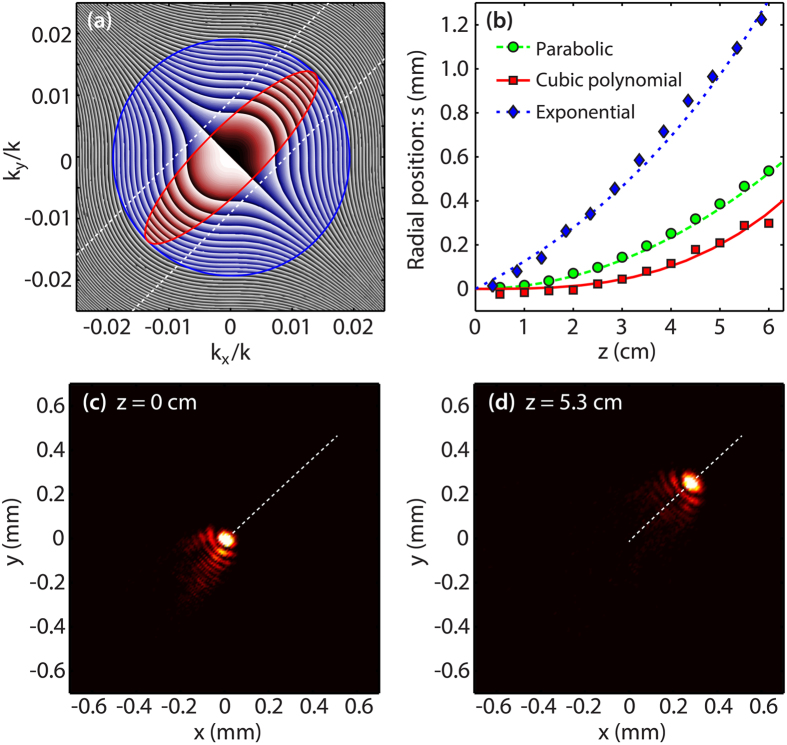
Propagation characteristics of self-accelerating beams initiated from an elliptical beam. (**a**) Phase mask applied on the SLM for a parabolic trajectory and corresponding beam intensity overlap at 95% cutoff for an elliptical incident Gaussian beam (red shading) reshaped from its circular counterpart (blue shading). (**b**) Radial position of the main hump as a function of propagation obtained for the three convex trajectories. Lines and markers show, respectively, the analytical and experimental results. (**c–d**) Experimental transverse intensity maps at *z* = 0 and *z* = 5.3 cm for the case of the parabolic trajectory.

Moreover, several related applications of these beams may indeed require an optimal spatial confinement of the beam intensity (e.g., pump-probe measurements or optical mapping), an important requirement that is usually hampered by several experimental factors such as the numerical apertures or the spatial resolutions of the optical elements employed. A practical and straightforward method that can be used to achieve this intensity enhancement (i.e., confinement) is to reshape the incident circular (Gaussian) beam into an elliptical (Gaussian) beam, whose minor diameter (orthogonal to the *k_s_*-axis and noted *ds*’) matches closely the stripe width determined by the spectrum of the main lobe (see Fig. 1(c)). This approach is illustrated in Fig. 2(a) where the initial circular Gaussian beam (blue shading) incident on the SLM phase mask has been reshaped into an elliptical Gaussian beam (red shading) by using a one-dimensional telescope (i.e., two conjugated cylindrical lenses of focal lengths f_1 _= 200 mm and f_2 _= 50 mm, respectively). In the second case, presented in Fig. 2(b), the experimentally recorded acceleration profiles for all studied convex trajectories (markers) remained almost unchanged and in excellent agreement with the numerical predictions (lines). The corresponding experimental transverse intensity maps generated from the elliptical beam considering the case of the parabolic trajectory are shown, as an illustrative example, in Fig. 2(c–d). The beam profile, similar to those of zero-order accelerating parabolic beams^17^,^18^, exhibits smaller side lobes compared to the uncompressed case presented in Fig. 1(a) and one would thus expect the beam peak intensity to be enhanced in terms of energy conservation. Interestingly, such a beam propagates without any significant deformation while following the expected acceleration profile.

### Characterization of the Peak Intensity Enhancement

**Figure 3:**
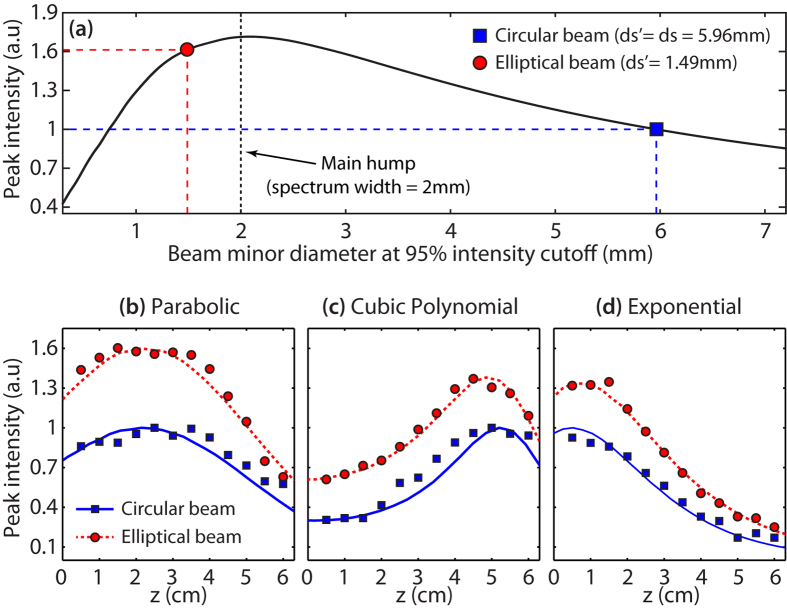
Peak intensity enhancement relative to the input beam asymmetry. (**a**) Expected peak intensity enhancement for the parabolic trajectory case calculated as a function of the minor diameter (*ds*’) of the elliptical Gaussian beam. The major diameter is constrained to the experimental value of *ds* = 5.96 mm. (**b–d**) Measured peak intensities as a function of the longitudinal distance obtained for the trajectories under investigation. The results obtained in the case of an elliptical (red) and a circular (blue) incident beam are compared. Lines and markers respectively show simulation fittings and experimental results.

In order to explore the effect of beam “squeezing” in relation to the peak intensity enhancement, we have performed numerical simulations for the case of the parabolic trajectory, which are shown in Fig. 3(a). According to these, we expect our experimental elliptical beam (red circle) to provide an enhancement of the peak intensity of about 60% when compared to the initial circular Gaussian beam (blue square). As illustrated in Fig. 3(a), the amount of “squeezing” of the input beam will determine the expected peak intensity enhancement and is intrinsically related to the associated numerical aperture of the system. For instance, considering the experimental setup described in this paper, one would expect the energy harvesting to be optimal whenever the beam spectrum exhibits a maximal overlap with the main lobe spectral components of the associated trajectory (i.e. for a beam minor diameter of 2 mm in this particular case). Obviously, further increasing the eccentricity of the beam shape would in fact be detrimental as one would basically approach the case of a one-dimensional (1D) beam (when the value of the minor diameter decreases largely). In this framework, and to confirm the validity of our numerical predictions, we have also compared the peak intensity evolution along propagation for the uncompressed and the compressed cases. These results, obtained experimentally for the trajectories given in Table 1, are presented in Fig. 3(b–d). For instance, the case of the parabolic trajectory is shown in Fig. 3(b) by comparing the peak intensity for a circular (blue squares) and an elliptical (red circles) incident beam. We can see a very good agreement with the 60% intensity enhancement expected from the simulations, while the overall longitudinal evolution of the peak intensity shows a similar behavior in both cases. For the cases of cubic and exponential trajectories presented in Fig. 3(c,d), respectively, one can also observe an enhancement of the peak intensity approximating 30%, still providing an excellent agreement with simulations.

**Figure 4:**
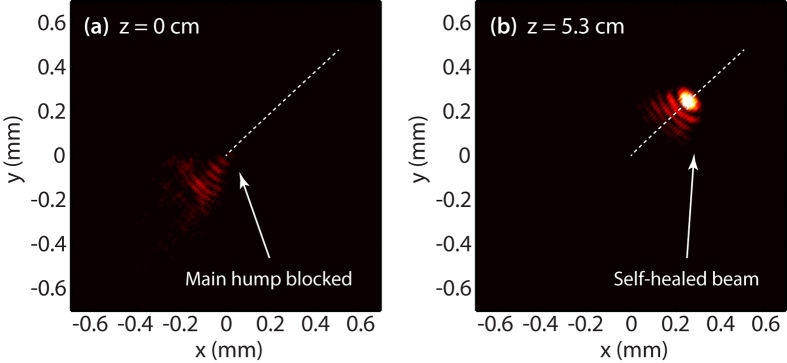
Experimental results illustrating the self-healing of a 2D accelerating beam initiated from an elliptical beam following a parabolic trajectory. Transverse intensity maps at (**a**) *z* = 0, where the main hump has been blocked at the onset of propagation, and (**b**) *z* = 5.3 cm. The parameters for the beam are the same as those of Fig. 2(c–d).

Intuitively, one may infer that the reduced sub-lobes would limit the self-healing over only a limited longitudinal range for the newly generated accelerating beams. Nonetheless, we did not observe significant changes in this important property before and after the compression of the spatial spectrum. In the experiment, we verified this issue by blocking the main lobe at the propagation onset (z = 0). The result obtained in this case is shown in Fig. 4 where one can directly observe the self-healing behavior of the beam associated to the parabolic trajectory illustrated in Fig. 2(c–d). This result further highlights the capability of our approach to provide energetically confined beam patterns while retaining the peculiar properties of 2D accelerating beams.

## Discussion

The scheme discussed in this report demonstrates the possibility to significantly and efficiently enhance the peak intensity of the main lobe of 2D diffraction-free beams propagating along several convex trajectories while reducing their equivalent transverse expansion. This energy confinement can be readily obtained by reshaping the incident beam to properly match the profile of the spectra associated to the main lobe. Such a simple and realistic approach is very useful since power is always an important concern in various applications of nonlinear optics, micro or nano-manipulation, laser writing, and ultra-intense field optics, where accelerating beams targeting various applications have been recently implemented. By using our method, one can achieve the same results previously demonstrated but using much lower input powers (more than 60% less than those required when employing conventional self-accelerating beams). Further work will aim at extending this scheme to the case of accelerating beams propagating in a nonlinear medium as well as studying optimal energy confinement in their spatio-temporal analog (i.e., 2 + 1 D optical bullets)^20^.

## Methods

Initial experimental measurements were performed using an incident Gaussian beam (CW at *λ* = 633 nm, *w*_0 _= 2.45 mm) directly illuminating a phase-only Pluto Spatial Light Modulator (SLM) produced by Holoeye (Pluto −1920 × 1080 pixels of 8 × 8 μm^2^ area, 8-bit grey phase levels). The SLM was located in the Fourier plane of a spherical lens (*f* = 150 mm) and a CCD camera (Sony XC-ST50 −640 × 480 pixels of 8.4 × 9.8 μm^2^ area, 8-bit dynamic range) mounted on a translation stage was used to image the beam transverse patterns and corresponding spectral intensity distributions at different longitudinal distances Fig. 1(a) and Fig. 1(c). In the latter case, an adjustable aperture slit was used to filter out the contributions of the beam side-lobes and the residual components were imaged in the Fourier plane of a second spherical lens (*f* = 100 mm) also mounted on a translation stage. Beam trajectories and key spatial frequencies [in Figs. 1(b,d) and 2(b)] were extracted by numerical methods from the intensity beam patterns and spectral spot distributions, taking into account the magnification and the transverse spatial resolution of our imaging system. In the second set of measurements, the initial circular Gaussian beam was reshaped into an elliptical Gaussian beam by using a one-dimensional telescope (i.e., two conjugated cylindrical lenses of *f*_1 _= 200 mm and *f*_2 _= 50 mm) while ensuring that the overall transmitted power was the same in both cases.

Note that all figures illustrating the transverse intensity distributions (and the associated spectral maps) are presented using a color scale normalized with respect to the maximal intensity measured. This is done to provide a more visual illustration of the reduced beam expansion obtained when the input beam has been reshaped. Furthermore, the peak intensity enhancement shown in Fig. 3(b-d) has been normalized (without any adjustment) to the maximal peak intensity detected on the CCD, by considering the propagation of each beam trajectory for the circular Gaussian input case. As the overall power for both input beams (respectively circular and elliptical) has been carefully characterized to be the same at the input and output of the imaging system (through both power measurements and transverse spatial integration of the CCD signals), this approach gives us a direct and straightforward measurement of the peak intensity enhancement obtained by our method.

## Additional Information

**How to cite this article**: Bongiovanni, D. *et al*. Efficient Optical Energy Harvesting in Self-Accelerating Beams. *Sci. Rep*. **5**, 13197; doi: 10.1038/srep13197 (2015).

## Figures and Tables

**Table 1:** Parameters of the 2D self-accelerating beams under study.

Type of Trajectory	Trajectory [x, y] = f (z)	Key Spatial Frequency (k_xc _= k_yc_)
Parabolic	0.1 z^2^, 0.1 z^2^	0.2 kz
Cubic polynomial	1.13 z^3^, 1.13 z^3^	3.39 kz^2^
Exponential	4.10^−4^ (e^20z^ −1), 4.10^−4^ (e^20z^ −1)	8.10^−3 ^ke^20z^
